# Coexpression profile of leukemic stem cell markers for combinatorial targeted therapy in AML

**DOI:** 10.1038/s41375-018-0180-3

**Published:** 2018-06-26

**Authors:** S. Haubner, F. Perna, T. Köhnke, C. Schmidt, S. Berman, C. Augsberger, F. M. Schnorfeil, C. Krupka, F. S. Lichtenegger, X. Liu, P. Kerbs, S. Schneider, K. H. Metzeler, K. Spiekermann, W. Hiddemann, P. A. Greif, T. Herold, M. Sadelain, M. Subklewe

**Affiliations:** 1 Department of Medicine III, University Hospital, LMU Munich, Munich, Germany; 2 Translational Cancer Immunology, Gene Center, LMU Munich, Munich, Germany; 3 Center for Cell Engineering and Immunology Program, Memorial Sloan Kettering Cancer Center, New York, NY USA; 4 German Cancer Consortium (DKTK), Heidelberg, Germany; 5 German Cancer Research Center (DKFZ), Heidelberg, Germany

**Keywords:** Acute myeloid leukaemia, Cancer immunotherapy, Targeted therapies, Cancer stem cells, Immunotherapy

## Abstract

Targeted immunotherapy in acute myeloid leukemia (AML) is challenged by the lack of AML-specific target antigens and clonal heterogeneity, leading to unwanted on-target off-leukemia toxicity and risk of relapse from minor clones. We hypothesize that combinatorial targeting of AML cells can enhance therapeutic efficacy without increasing toxicity. To identify target antigen combinations specific for AML and leukemic stem cells, we generated a detailed protein expression profile based on flow cytometry of primary AML (*n* = 356) and normal bone marrow samples (*n* = 34), and a recently reported integrated normal tissue proteomic data set. We analyzed antigen expression levels of CD33, CD123, CLL1, TIM3, CD244 and CD7 on AML bulk and leukemic stem cells at initial diagnosis (*n* = 302) and relapse (*n* = 54). CD33, CD123, CLL1, TIM3 and CD244 were ubiquitously expressed on AML bulk cells at initial diagnosis and relapse, irrespective of genetic characteristics. For each analyzed target, we found additional expression in different populations of normal hematopoiesis. Analyzing the coexpression of our six targets in all dual combinations (*n* = 15), we found CD33/TIM3 and CLL1/TIM3 to be highly positive in AML compared with normal hematopoiesis and non-hematopoietic tissues. Our findings indicate that combinatorial targeting of CD33/TIM3 or CLL1/TIM3 may enhance therapeutic efficacy without aggravating toxicity in immunotherapy of AML.

## Introduction

Despite some advances in the treatment of acute myeloid leukemia (AML) in recent years, overall prognosis remains poor [1]. Allogeneic stem cell transplantation is still the only curative option in high risk and relapsed AML, but morbidity and mortality are high owing to transplant-related side effects and refractory disease [2]. Targeted immunotherapy provides a potent option to specifically eliminate chemoresistant leukemic stem cells, which are reported to be the main cause of relapse [3]. In relapsed B-cell acute lymphoblastic leukemia (B-ALL), targeting of CD19 via bispecific T-cell-engaging antibody constructs and chimeric antigen receptor (CAR) T-cell products has shown remarkable antileukemic effects and a tolerable safety profile [4–10]. Given its success in B-ALL, the translation of T cell-based targeted immunotherapy to AML is of major interest and currently being evaluated in preclinical and clinical trials. However, choice of suitable target antigens in AML has proven to be challenging.

In B-ALL, CD19 can be considered an ideal target antigen owing to its high expression on leukemic cells and its restricted expression profile on normal cells: it is expressed on essentially all B-lineage cells, whereas negative on all other hematopoietic lineages or other normal tissues [11, 12]. In line with the CD19 expression pattern, the on-target off-leukemia toxicity of CD19 CAR T-cell therapy is tolerable and limited to B-cell aplasia [13]. In AML, however, the most suitable target antigen still needs to be defined. Several surface-bound target antigens are known to be overexpressed on AML cells [14–32]. Some of these, including CD33, CD123, CLL1, CD47, CD96, CD157, CD244, TIM3 and CD7, have been reported to be expressed on leukemic stem cells (LSC) [20, 21, 24, 27, 32–36]. However, single-targeting approaches against these LSC-associated antigens is complicated as none of the antigens are exclusively expressed on AML cells, leading to severe on-target off-leukemia toxicity [37–40]. Furthermore, clonal heterogeneity or antigen escape mechanisms could lead to persistence of AML cells upon single-targeting therapy.

Perna et al. [12] recently provided the rationale that a combinatorial targeting approach with well-matched target antigens could have the potential to enhance therapeutic efficacy without increasing on-target off-leukemia toxicity. In our study, we analyzed the coexpression profile of the most commonly targeted and leukemic stem cell-associated antigens within a single cohort of > 300 AML patients, comparing primary AML cells to normal hematopoietic cells and non-hematopoietic tissues. We sought to identify combinations of target antigens that show high coexpression in AML compared with normal cells and thereby provide options to reduce toxicity and overcome antigen escape mechanisms as well as clonal heterogeneity in AML.

## Materials and methods

### Patient samples and clinical data

For flow cytometric analysis, peripheral blood or bone marrow aspirate samples of AML patients at initial diagnosis (*n* = 302) and relapse (*n* = 54) as well as bone marrow aspirates from healthy donors (*n* = 34) were used. Sample size was chosen based on feasibility and experience with previous analysis [34]. All samples were collected after written informed consent in accordance with the Declaration of Helsinki and approval by the Institutional Review Board of the Ludwig Maximilian University Munich. Patient characteristics are summarized in Table 1. Diagnostic workup to establish diagnosis of AML included cytomorphology, cytogenetics, fluorescence in situ hybridization, molecular genetics and immunophenotyping. Combined cytogenetic and molecular risk stratification groups were assigned in accordance with the Medical Research Council (MRC) and European LeukemiaNet (ELN) recommendations [41, 42].

**Table 1:** Patient characteristics of primary AML samples for flow cytometric analysis

	All cases	Initial Dx	Relapse
Samples, *n*	356	302	54
Age, median (range)	63 (16–92)	65 (16–92)	55 (23–80)
Gender, *n* (%)			
Male	201 (56%)	170 (56%)	31 (57%)
Female	155 (44%)	132 (44%)	23 (43%)
FAB, *n* (%)			
M0	18 (8%)	12 (7%)	6 (19%)
M1	68 (32%)	61 (34%)	7 (23%)
M2	45 (21%)	40 (22%)	5 (16%)
M3	9 (4%)	8 (4%)	1 (3%)
M4	41 (19%)	35 (19%)	6 (19%)
M5	30 (14%)	24 (13%)	6 (19%)
M6	2 (1%)	2 (1%)	0 (0%)
M7	0 (0%)	0 (0%)	0 (0%)
Unknown	143	120	23
Cytogenetics, *n* (%)			
Normal karyotype	139 (43%)	123 (44%)	16 (36%)
Complex karyotype	73 (23%)	60 (21%)	13 (30%)
t(8;21)	9 (3%)	9 (3%)	0 (0%)
t(9;11)(p21-22;q23) or t(11;19)(q23;p13)	10 (3%)	9 (3%)	1 (2%)
inv(16)/t(16;16)(p13;q22)	9 (3%)	9 (3%)	0 (0%)
Other adverse risk abnormalities	22 (7%)	17 (6%)	5 (11%)
Non-classified abnormalities	62 (19%)	53 (19%)	9 (20%)
Unknown	32	22	10
Mutations (normal karyotype), *n* (%)			
NPM1 mut/FLT3 wt	38 (28%)	37 (31%)	1 (6%)
NPM1 mut/FLT3-ITD	35 (26%)	30 (25%)	5 (31%)
NPM1 wt/FLT3-ITD	14 (10%)	14 (12%)	0 (0%)
NPM1 wt/FLT3 wt	50 (36%)	40 (33%)	10 (63%)
CEBPA mut	6 (10%)	6 (10%)	0 (0%)
KMT2A mut	16 (12%)	12 (10%)	4 (22%)
MRC, *n* (%)			
Favorable	46 (14%)	44 (16%)	2 (4%)
Intermediate	178 (55%)	154 (55%)	24 (53%)
Adverse	100 (31%)	81 (29%)	19 (42%)
Unknown	32	23	9
ELN2010, *n* (%)			
Favorable	64 (20%)	62 (23%)	2 (4%)
Intermediate-I	90 (28%)	74 (27%)	16 (36%)
Intermediate-II	64 (20%)	55 (20%)	9 (20%)
Adverse	100 (31%)	82 (30%)	18 (40%)
Unknown	38	29	9

### Flow cytometry

After collection, all samples were analyzed immediately, without prior cryoconservation. Samples were stained with the following fluorochrome-conjugated anti-human monoclonal antibodies: CD7 (clone 8H8.1, Beckman Coulter, #A97050), CD33 (clone D3HL60.251, Beckman Coulter, #A54824), CD34 (clone 581, Beckman Coulter, #B49202), CD38 (clone LS198.4.3, Beckman Coulter, #B49200), CD45 (clone J.33, Beckman Coulter, #B36294), CD123 (clone 7G3, BD, #560087), CD244 (clone C1.7, Biolegend, #329508), TIM3 (clone 344823, R&D, #FAB2365A). Corresponding isotype controls were used for each sample. Surface antigen expression was assessed using a 10-color Navios flow cytometer (Beckman Coulter, Brea, CA, USA). Gating was performed as described in Supplemental Fig. 1A. As measure of antigen expression intensity, the median fluorescence intensity (MFI) ratio was used. MFI ratio was calculated by dividing the MFI value of the antigen-specific antibody by the MFI value of the respective isotype control (Supplemental Fig. 1B), as previously described [34]. We compared the isotype-based MFI ratio with an alternative MFI index, which is based on normalization to lymphocytes (Supplemental Fig. 2). The MFI ratio highly correlated with the MFI index for the myeloid-associated antigens CD33, CD123 and CLL1 (Spearman *r* > 0.88). In contrast, we observed no or lower correlation of the MFI ratio with the MFI index for the lymphoid-associated antigens TIM3, CD244 and CD7 (Spearman *r* < 0.79). The latter was owing to expression of these antigens on lymphocytes, which therefore did not serve as appropriate negative control. For this reason, we chose the isotype-based MFI ratio for our analysis. Positive expression in the majority of cells was defined as MFI ratio ≥ 1.5. MFI values were determined using FlowJo software (Version 9.8.5) (Tree Star Inc., Ashland, Oregon).

### Normal tissue proteomics

Protein expression data for normal tissues were retrieved from the integrated dataset generated by Perna et al. [12], including three independent protein expression data repositories: the Human Protein Atlas, the Human Proteome Map and the Proteomics Database.

### Statistical analysis

Statistical analysis was performed using GraphPad Prism 7 (GraphPad Software, Inc., La Jolla, USA). The significance of differences was determined using the Mann–Whitney *U* test for unpaired samples and the Wilcoxon matched-pairs signed rank test for paired samples. Statistical significance was considered for *p* < 0.05 (*), *p* < 0.01 (**), *p* < 0.001 (***) and *p* < 0.0001 (****). Results are shown as medians ± 95% confidence interval or as indicated. Graphs were generated using GraphPad Prism 7, R Studio (R Studio, Boston, USA) and Adobe Illustrator CS6 (Adobe Systems, San José, USA).

## Results

### Antigen expression on AML cells at initial diagnosis and at relapse

**Fig. 1:**
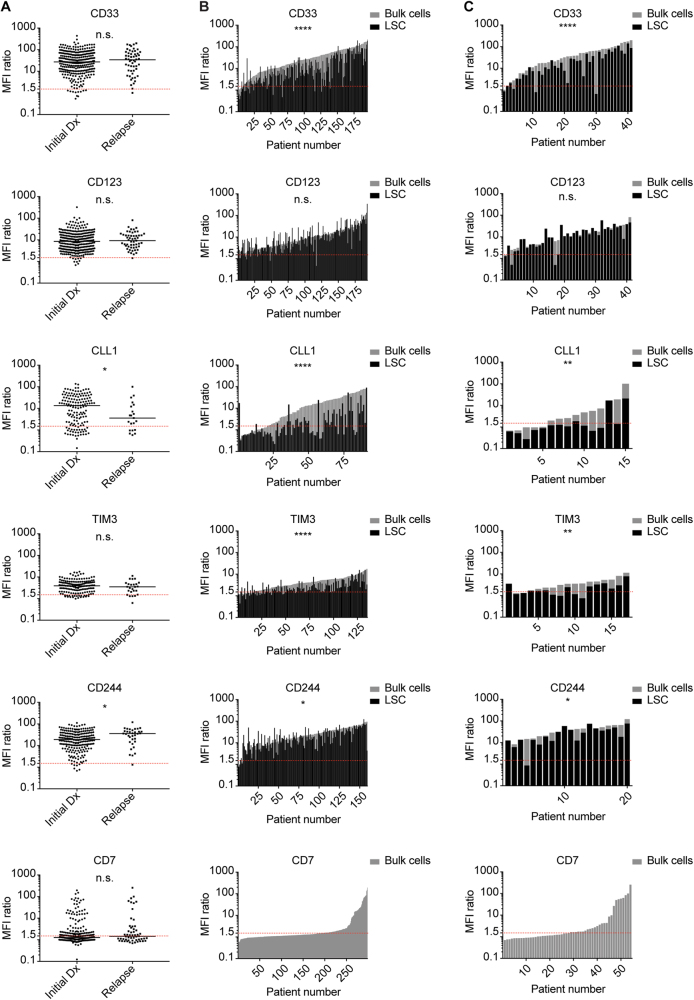
Antigen Expression on AML Bulk Cells and LSC at Initial Diagnosis and Relapse. Antigen expression (MFI ratio) on primary AML samples at initial diagnosis and relapse was determined via flow cytometry. Each dot or bar represents one patient sample. Red dotted line indicates MFI ratio of 1.5 as cutoff for positivity. **a** Initial diagnosis vs. relapse: unpaired analysis of AML bulk cells. **b** Initial diagnosis: paired analysis of AML bulk cells (gray) and LSC (black). **c** Relapse: paired analysis of AML bulk cells (gray) and LSC (black)

To analyze the expression profile of AML-associated surface antigens, we performed multicolor flow cytometry on primary AML samples at initial diagnosis (*n* = 302) and at relapse (*n* = 54). We quantified the antigen expression levels of CD33, CD123, CLL1, TIM3, CD244 and CD7 on AML bulk cells (as described in Supplemental Fig. 1). Antigens were considered positive in the majority of cells if expression intensity exceeded an MFI ratio of 1.5. At initial diagnosis, AML bulk cells in most patients were positive for CD33 (96.4%), CD123 (97.0%), CLL1 (80.1%), TIM3 (87.3%) and CD244 (96.7%). Also at relapse, AML bulk cells in most patients were positive for CD33 (98.1%), CD123 (98.1%), CLL1 (71.4%), TIM3 (80.0%) and CD244 (97.1%). The aberrant antigen CD7 was positive in 35.6% of the patients at initial diagnosis and 48.1% of the patients at relapse (Fig. 1a, Supplemental Table 1). Both at initial diagnosis and at relapse, our analysis did not show any correlation of antigen expression levels with patient age (Supplemental Fig. 3). Subgroup analysis of de novo vs. secondary AML did not find any significant differences in expression levels of CD33, CD123, CLL1, TIM3 and CD7. In contrast, CD244 was found to be significantly higher in sAML after MDS compared with de novo AML (*p* = 0.02) (Supplemental Fig. 4). In addition, we analyzed the expression level of antigens in relapsed AML after intensive chemotherapy alone (*n* = 33) and after allogeneic stem cell transplantation (*n* = 15) (Supplemental Figure 5). In this small subgroup analysis, antigen expression levels of CD33, CD123, CD244 and CD7 were not significantly different after intensive chemotherapy alone compared with allogeneic stem cell transplantation. Statistical analysis of TIM3 and CLL1 expression levels was not performed owing to low sample numbers.

### Antigen expression on AML bulk cells and leukemic stem cells

On AML bulk cells, we found varying degrees of surface antigen density both at initial diagnosis and at relapse, hereafter quantified as median MFI ratios (Fig. 1a, Supplemental Table 1; negative: < 1.5; low: 1.5–5; medium: 5–15; high: > 15): High expression was found for CD33 (Initial diagnosis: 27.1/Relapse: 34.1) and CD244 (18.9/35.8). Moderate expression was found for CD123 (8.5/9.2), CLL1 (13.5/3.6) and TIM3 (3.9/3.5). Median expression of CD7, which is known to be aberrantly expressed in AML, was negative per our definition. Comparing antigen expression levels at initial diagnosis and at relapse, no significant differences were found for CD33, CD123, TIM3 and CD7. CLL1 expression was significantly lower at relapse, whereas CD244 was significantly higher at relapse. The same trend could be observed in matched-pair analysis at initial diagnosis and relapse, however, without statistical significance that may owe to low sample numbers (Supplemental Figure 6). We next evaluated antigen expression on LSC. At initial diagnosis, LSC in most patients were positive for CD33 (88.7%), CD123 (95.3%), TIM3 (78.5%) and CD244 (98.1%) (Fig. 1b, Supplemental Table 1). Also at relapse, LSC in most patients were positive for CD33 (90.2%), CD123 (92.7%), TIM3 (64.7%) and CD244 (95.0%) (Fig. 1c, Supplemental Table 1). In contrast, CLL1 expression on LSC showed interindividual variability, with only a subgroup of patients being positive at initial diagnosis (45.1%) and at relapse (20.0%). Comparing antigen expression levels on AML bulk cells and LSC, we found CD33, CLL1, TIM3 and CD244 to be significantly less expressed on LSC, both at initial diagnosis and at relapse. In contrast, we found a trend toward higher CD123 expression on LSC compared with bulk cells, both at initial diagnosis and at relapse (*p* = 0.05 and *p* = 0.08, respectively) (Fig. 1b, c, Supplemental Table 1).

### Antigen expression in genetically defined AML subgroups

To verify potential correlations of AML-associated antigens with genetic characteristics, we analyzed the level of antigen expression in different cytogenetically and molecularly defined AML risk groups according to the MRC and ELN2010 criteria (Supplemental Figure 7A). ELN2010 favorable and adverse risk AML samples did not have significantly different antigen expression levels of CD33, CD123, CLL1, TIM3, CD244 and CD7. We next analyzed antigen expression in different molecularly defined subtypes of AML. Normal karyotype AML samples were divided into sub-cohorts based on the mutational status of NPM1, FLT3, CEBPA and KMT2A (Supplemental Figure 7B). NPM1 mut/FLT3 wt (*n* = 37), NPM1 mut/FLT3-ITD (*n* = 30) and NPM1 wt/FLT3-ITD (*n* = 14) AML samples each had significantly higher expression of CD33 and CD123 compared with NPM1 wt/FLT3 wt (*n* = 40) AML samples, indicating that mutations of NPM1 and FLT3 are independently associated with higher expression of CD33 and CD123 compared with AML with non-mutated NPM1 or FLT3, respectively. Of note, in all evaluated molecularly defined sub-cohorts CD33, CD123, CLL1, TIM3 and CD244 were positive in most samples.

### Antigen expression on AML cells and normal hematopoietic cells

**Fig. 2:**
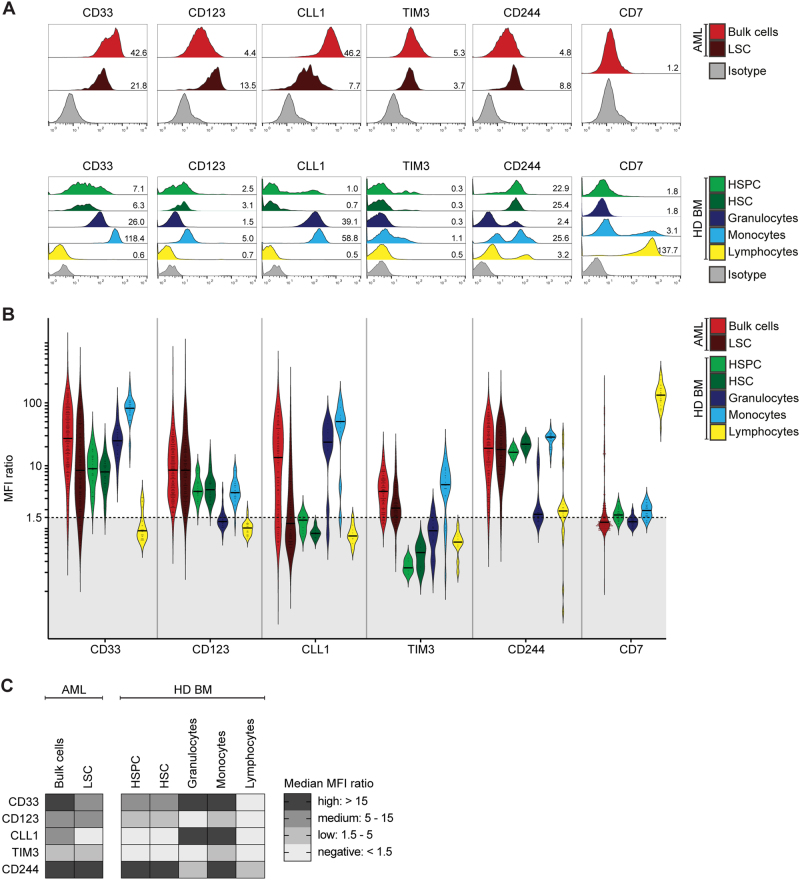
Antigen Expression in AML and Normal Hematopoiesis. Antigen expression on primary AML samples at initial diagnosis and healthy donor-derived bone marrow cell populations (HD BM) as indicated in legend. **a** Representative primary AML sample and healthy donor-derived bone marrow sample. Histograms indicate fluorescence intensity. Numbers indicate MFI ratio. **b** Antigen expression in AML and normal hematopoiesis, shown as MFI ratio. Dots indicate measured samples. Violin plots illustrate distribution of antigen expression for each analyzed cell population. Black dotted line indicates MFI ratio of 1.5 as cutoff for positivity. **c** Summary of antigen expression levels (median MFI ratio)

As RNA expression of CD33, CD123, CLL1, TIM3, CD244 and CD7 was similar in bone marrow of AML patients at initial diagnosis and complete remission (Supplemental Figure 8, Supplemental Table 4), we next evaluated the differential protein expression on AML bulk cells and LSC in comparison with healthy donor-derived hematopoietic stem/progenitor cells (HSPC/HSC), granulocytes, monocytes and lymphocytes (representative measurements in Fig. 2a; gating in Supplemental Fig. 1A). Overall, HSPC were positive for CD33 and CD123, but negative for CLL1 and TIM3. Granulocytes were positive for CD33, CLL1 and CD244, but negative for CD123 and TIM3. Monocytes were positive for all tested antigens. Lymphocytes were only positive for CD244 and CD7 (Fig. 2, Supplemental Table 2). This heterogeneous expression pattern both in the stem/progenitor and differentiated compartments implied different on-target off-leukemia hematotoxicity profiles for each of our tested target antigens.

### Combinatorial antigen expression on AML cells and normal hematopoietic cells

**Fig. 3:**
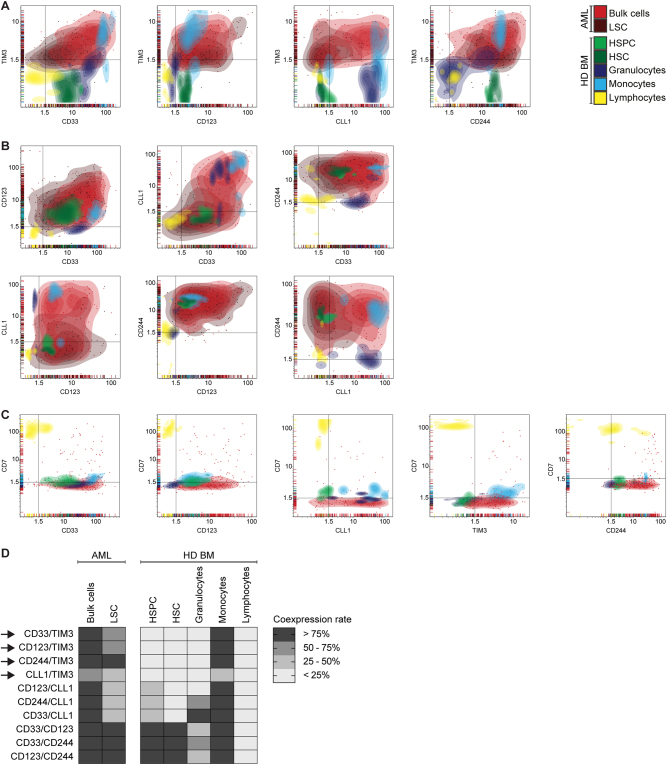
Antigen Coexpression in AML and Normal Hematopoiesis. Antigen coexpression on primary AML cells and on normal hematopoietic cells of healthy donor-derived bone marrow (HD BM). Each dot indicates one independent sample measurement and the MFI ratios for the respective pair of antigens. Colors indicate different cell populations. Colored areas indicate density of dots. Black lines indicate MFI ratio of 1.5 as cutoff for positivity. Coexpression rate indicates the proportion of samples with antigen coexpression (MFI ratio > 1.5 for both antigens) in relation to all measured samples. **a** Suitable target antigen combinations. **b** Unsuitable target antigen combinations. **c** Target antigen combinations including CD7. **d** Summary of antigen coexpression data. Arrows indicate suitable target antigen combinations

We hypothesized that combinatorial targeting of antigens with non-overlapping expression on normal cells can enhance therapeutic efficacy without increasing toxicity. To identify the most AML-specific dual antigen combinations, we analyzed the antigen coexpression profile of CD33, CD123, CLL1, TIM3, CD244 and CD7 in primary AML compared with healthy donor-derived bone marrow samples, which were analyzed separately (Fig. 3). To systematically screen for suitable target antigen combinations, we calculated for each cell population the percentage of samples with dual antigen positivity (MFI ratio ≥ 1.5 for both antigens) and then compared antigen coexpression in AML vs. normal hematopoiesis (Supplemental Table 3). Suitable target antigen combinations were defined as having antigen coexpression on HSPC in 0% of samples and on granulocytes and/or lymphocytes in < 25% of samples. Combinations of the most commonly targeted antigens, CD33/CD123, CD33/CLL1 and CLL1/CD123, did not fulfill these criteria, owing to high coexpression on hematopoietic stem/progenitor cells (Fig. 3b). Remarkably, target antigen combinations that included TIM3 were found to have a more suitable coexpression profile with absent coexpression on HSPC and granulocytes (Fig. 3a). Thus, we identified CD33/TIM3, CD123/TIM3, CLL1/TIM3 and CD244/TIM3 as suitable target antigen combinations based on the expression profile in AML and normal hematopoiesis. Coexpression on single cell level could be confirmed both on AML bulk cells and LSC (Supplemental Figure 9).

### Combinatorial antigen expression in normal non-hematopoietic tissue

**Fig. 4:**
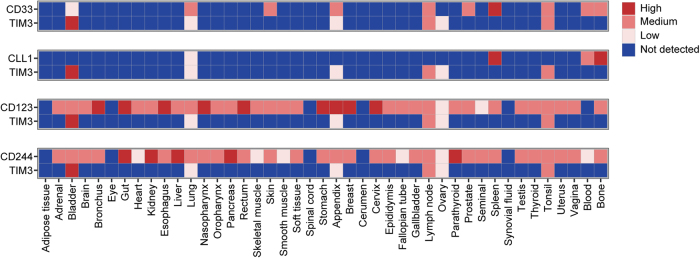
Antigen coexpression in normal tissues. Protein expression levels in normal tissues were defined by using a previously reported integrated dataset. Paired expression is illustrated for possible target antigen combinations CD33/TIM3, CLL1/TIM3, CD123/TIM3 and CD244/TIM3. Protein expression data range from 0 (not detected) to 3 (high)

To evaluate whether the selected target combinations were coexpressed on other, non-hematopoietic cells, we analyzed protein expression of CD33, CD123, CLL1, TIM3 and CD244 in normal tissues by using the integrated data set reported by Perna et al. [12] (Fig. 4). Based on these data, CD123 and CD244 were found to be expressed in a broad range of non-hematopoietic tissues, including several vital organs: high positivity was detected for CD123 in lung and gut and for CD244 in gut, liver and kidney. In contrast, CD33, TIM3 and CLL1 showed an expression pattern that was largely restricted to hematopoietic tissue or organs with high immune cell infiltration. Combinatorial analysis of CD33/TIM3 and CLL1/TIM3 revealed non-overlapping expression patterns in normal tissues: Excluding organs with known immune infiltration, dual expression of CD33/TIM3 was only found in bladder. Remarkably, for CLL1/TIM3, there was no dual expression in any tissue except for low expression levels in lung.

Starting from our panel of six AML-associated antigens and all possible dual antigen combinations thereof (*n* = 15), our analysis revealed that CD33/TIM3 and CLL1/TIM3 were coexpressed in most AML samples, but largely spared in normal hematopoiesis and non-hematopoietic tissues, thus meeting our criteria of suitable target antigen combinations.

## Discussion

In this study, we provide a detailed cell surface protein expression analysis of six commonly targeted and leukemic stem cell-associated antigens in AML. For the first time, we directly compare the antigen expression levels and coexpression of CD33, CD123, CLL1, TIM3, CD244 and CD7 on AML bulk cells, LSC and normal bone marrow cells, based on a cohort of > 300 AML patients and 34 healthy donors.

We show that CD33, CD123 and CLL1 are highly expressed on AML cells of most patients, which is consistent with previous reports [14, 21, 34, 43]. Notably, we are the first to compare the antigen expression levels on AML bulk cells and LSC at initial diagnosis and relapse. As multiple clinical trials are currently targeting CD33 and CD123 in relapsed and refractory (r/r) AML, our data support the target suitability by showing homogeneous expression of CD33 and CD123 at relapse. Yet, consistent with independent data from our previous report, we show significantly lower expression of CD33 on LSC compared to bulk cells, underlining the difficulty to specifically target LSC [34]. In comparison, we find CD123 to be more specifically overexpressed on AML cells, but positivity on hematopoietic stem and progenitor cells as well as high expression in multiple normal tissues could lead to on-target toxicity, which may explain the preclinically observed hematotoxicity and the clinically reported grade 4 and 5 events upon CD123-targeting therapy [12, 37, 44, 45]. For CLL1, we find positivity on AML bulk cells in most cases, but lower and interindividually variable expression on LSC suggests that CLL1 is not a universal LSC marker. This is in agreement with a previous report stating CLL1 as a useful marker for LSC but also describing a heterogeneous expression pattern [35]. Given the limited CLL1 expression on normal HSPC and non-hematopoietic tissues, CLL1 may be a preferable target in a selected subgroup of patients. For CLL1-targeting therapy in AML, selection of patients based on the individual level of CLL1 expression may be necessary.

Beside the commonly targeted AML-associated antigens, CD244 and TIM3 have been reported to be overexpressed on LSC and to have a direct leukemia-promoting effect by maintaining the proliferative capability of LSC [22, 32, 46–50]. In our study, we show ubiquitous CD244 expression on AML bulk cells and LSC. However, high CD244 expression on HSPC and monocytes as well as in several vital non-hematopoietic tissues suggest that CD244 is a very unspecific AML-associated antigen. On the contrary, we describe a more suitable expression profile for TIM3, with positivity on AML bulk cells and LSC in most patients and negativity in the majority of normal HSPC, granulocytes, lymphocytes and most normal non-hematopoietic tissues. However, small subpopulations with variations in antigen expression level, e.g., TIM3-positive T cells, might be underrepresented in our study. In the context of immune activation, upregulation of TIM3 on activated and exhausted T cells could result in on-target toxicity with depletion of TIM3-positive normal T cells [51]. We previously reported that TIM3 is expressed on < 5% of peripheral blood T cells in AML patients at initial diagnosis and relapse [52]. The relevance of TIM3-directed toxicity of CAR T cells against TIM3-positive normal T cells needs to be evaluated. However, TIM3-specific CAR T-cell fratricide or suicide may be limited in a CAR setting with optimized transgene delivery, thereby reducing exhaustion and TIM3 expression to a minor percentage of CAR T cells [53]. In addition, targeted deletion of the TIM3 gene may prevent CAR T-cell fratricide. Beside its relatively suitable expression profile on normal cells, TIM3 is part of an autocrine stimulatory loop that promotes self-renewal of LSC and thereby progression of AML [46]. Considering the pro-leukemic function of TIM3, targeting TIM3 may be less prone to escape via antigen loss and may enable specific elimination of LSC.

Overall, our protein expression data indicate that single-targeting of CD33, CD123, CLL1, TIM3 or CD244 may have antileukemic efficacy in most AML patients. However, on-target off-leukemia toxicity both in hematopoietic and non-hematopoietic tissues as well as high risk of relapse owing to antigen escape and clonal heterogeneity may limit therapeutic success. Among all tested antigens, we report a suitable expression pattern for TIM3, which may qualify for single-targeting or combinatorial targeting approaches in AML.

We hypothesize that combinatorial targeting strategies with well-matched target antigens enhance therapeutic efficacy without increasing on-target off-leukemia toxicity. Several preclinical reports of combinatorial targeting have been published so far and are either based on triplebodies or combinatorial CAR approaches [54–57]. In a combinatorial CAR setting with coexpression of two CARs (CAR + CAR), T cells eliminate any cells expressing at least one of the two targets, thereby reducing the chance of antigen escape. In a setting with coexpression of a CAR and a chimeric costimulatory receptor (CAR + CCR), T cells only eliminate cells that coexpress both targets, thereby limiting cytotoxicity to double-positive tumor cells and relatively sparing single-positive normal tissue [55]. For both approaches, our study defines suitable AML-associated target antigen combinations with non-overlapping expression patterns in normal cells. Out of *n* = 15 possible target antigen pairs, we identify CD33/TIM3 and CLL1/TIM3 as the most suitable antigen combinations, with high coexpression in most AML samples and largely absent coexpression in normal hematopoiesis and non-hematopoietic tissues, excluding tissues with known immune infiltration. Notably, targeting of CD33/TIM3 and CLL1/TIM3 may lead to monocyte depletion. Although the tolerability of monocyte depletion needs to be further evaluated, there may be a rationale for targeting not only AML cells but also monocytes that are involved in disease pathogenesis and progression [58]. The target antigen combination CD33/TIM3 could be particularly suitable for combinatorial CAR + CCR approaches, thereby minimizing stem cell and myeloid hematotoxicity and prioritizing LSC-targeting. However, this setting might facilitate immune escape of single-positive AML cells. Targeting CLL1/TIM3 could be feasible not only in the CAR + CCR setting, but also in CAR + CAR approaches, thereby maximizing the number of targetable AML cells and minimizing chances of antigen escape.

In summary, our comprehensive analysis of > 300 primary AML samples demonstrates antigen positivity of CD33, CD123, CLL1, TIM3 and CD244 in most cases at initial diagnosis and relapse, irrespective of the genetic background. While none of these antigens are truly AML-specific, we describe a suitable expression profile of TIM3 with limited expression on normal cells. Our coexpression analysis of hematopoietic cells and non-hematopoietic tissues identifies CD33/TIM3 and CLL1/TIM3 as promising antigen combinations that should be validated in dual-targeting immunotherapeutic strategies.

## Electronic supplementary material

Supplemental Table 1

Supplemental Table 2

Supplemental Table 3

Supplemental Table 4

Supplemental Figure 1

Supplemental Figure 2

Supplemental Figure 3

Supplemental Figure 4

Supplemental Figure 5

Supplemental Figure 6

Supplemental Figure 7

Supplemental Figure 8

Supplemental Figure 9

## References

[1] Dohner H, Estey E, Grimwade D, Amadori S, Appelbaum FR, Buchner T (2017). Diagnosis and management of AML in adults: 2017 ELN recommendations from an international expert panel. Blood.

[2] Breems DA, Van Putten WL, Huijgens PC, Ossenkoppele GJ, Verhoef GE, Verdonck LF (2005). Prognostic index for adult patients with acute myeloid leukemia in first relapse. J Clin Oncol.

[3] Shlush LI, Mitchell A, Heisler L, Abelson S, Ng SWK, Trotman-Grant A (2017). Tracing the origins of relapse in acute myeloid leukaemia to stem cells. Nature.

[4] Kantarjian H, Stein A, Gokbuget N, Fielding AK, Schuh AC, Ribera JM (2017). Blinatumomab versus chemotherapy for advanced acute lymphoblastic leukemia. N Engl J Med.

[5] Davila ML, Riviere I, Wang X, Bartido S, Park J, Curran K (2014). Efficacy and toxicity management of 19-28z CAR T cell therapy in B cell acute lymphoblastic leukemia. Sci Transl Med.

[6] Lee DW, Kochenderfer JN, Stetler-Stevenson M, Cui YK, Delbrook C, Feldman SA (2015). T cells expressing CD19 chimeric antigen receptors for acute lymphoblastic leukaemia in children and young adults: a phase 1 dose-escalation trial. Lancet.

[7] Maude SL, Frey N, Shaw PA, Aplenc R, Barrett DM, Bunin NJ (2014). Chimeric antigen receptor T cells for sustained remissions in leukemia. N Engl J Med.

[8] Turtle CJ, Hanafi LA, Berger C, Gooley TA, Cherian S, Hudecek M (2016). CD19 CAR-T cells of defined CD4+:CD8+ composition in adult B cell ALL patients. J Clin Invest.

[9] Qasim W, Zhan H, Samarasinghe S, Adams S, Amrolia P, Stafford S (2017). Molecular remission of infant B-ALL after infusion of universal TALEN gene-edited CAR T cells. Sci Transl Med.

[10] Grupp SA, Kalos M, Barrett D, Aplenc R, Porter DL, Rheingold SR (2013). Chimeric antigen receptor-modified T cells for acute lymphoid leukemia. N Engl J Med.

[11] LeBien TW, Tedder TF (2008). B lymphocytes: how they develop and function. Blood.

[12] Perna F, Berman SH, Soni RK, Mansilla-Soto J, Eyquem J, Hamieh M (2017). Integrating proteomics and transcriptomics for systematic combinatorial chimeric antigen receptor therapy of AML. Cancer Cell.

[13] Paszkiewicz PJ, Frassle SP, Srivastava S, Sommermeyer D, Hudecek M, Drexler I (2016). Targeted antibody-mediated depletion of murine CD19 CAR T cells permanently reverses B cell aplasia. J Clin Invest.

[14] Bakker AB, van den Oudenrijn S, Bakker AQ, Feller N, van Meijer M, Bia JA (2004). C-type lectin-like molecule-1: a novel myeloid cell surface marker associated with acute myeloid leukemia. Cancer Res.

[15] Chung SS, Eng WS, Hu W, Khalaj M, Garrett-Bakelman FE, Tavakkoli M (2017). CD99 is a therapeutic target on disease stem cells in myeloid malignancies. Sci Transl Med.

[16] Coles SJ, Wang EC, Man S, Hills RK, Burnett AK, Tonks A (2011). CD200 expression suppresses natural killer cell function and directly inhibits patient anti-tumor response in acute myeloid leukemia. Leukemia.

[17] Diermayr S, Himmelreich H, Durovic B, Mathys-Schneeberger A, Siegler U, Langenkamp U (2008). NKG2D ligand expression in AML increases in response to HDAC inhibitor valproic acid and contributes to allorecognition by NK-cell lines with single KIR-HLA class I specificities. Blood.

[18] Gillissen MA, Kedde M, De Jong G, Yasuda E, Levie SE, Bakker A, et al. Tumor specific glycosylated CD43 is a novel and highly specific target for acute myeloid leukemia and myelodysplastic syndrome. Cancer Immunol Res. 2016; 4:Abstract nr A026.

[19] Griffin JD, Linch D, Sabbath K, Larcom P, Schlossman SF (1984). A monoclonal antibody reactive with normal and leukemic human myeloid progenitor cells. Leuk Res.

[20] Hosen N, Park CY, Tatsumi N, Oji Y, Sugiyama H, Gramatzki M (2007). CD96 is a leukemic stem cell-specific marker in human acute myeloid leukemia. Proc Natl Acad Sci USA.

[21] Jordan CT, Upchurch D, Szilvassy SJ, Guzman ML, Howard DS, Pettigrew AL (2000). The interleukin-3 receptor alpha chain is a unique marker for human acute myelogenous leukemia stem cells. Leukemia.

[22] Kikushige Y, Shima T, Takayanagi S, Urata S, Miyamoto T, Iwasaki H (2010). TIM-3 is a promising target to selectively kill acute myeloid leukemia stem cells. Cell Stem Cell.

[23] Konopleva M, Rissling I, Andreeff M (2000). CD38 in hematopoietic malignancies. Chem Immunol.

[24] Krupka C, Lichtenegger FS, Kohnke T, Bogeholz J, Bucklein V, Roiss M (2017). Targeting CD157 in AML using a novel, Fc-engineered antibody construct. Oncotarget.

[25] Kuchenbauer F, Kern W, Schoch C, Kohlmann A, Hiddemann W, Haferlach T (2005). Detailed analysis of FLT3 expression levels in acute myeloid leukemia. Haematologica.

[26] Legras S, Gunthert U, Stauder R, Curt F, Oliferenko S, Kluin-Nelemans HC (1998). A strong expression of CD44-6v correlates with shorter survival of patients with acute myeloid leukemia. Blood.

[27] Majeti R, Chao MP, Alizadeh AA, Pang WW, Jaiswal S, Gibbs KD (2009). CD47 is an adverse prognostic factor and therapeutic antibody target on human acute myeloid leukemia stem cells. Cell.

[28] Peinert S, Prince HM, Guru PM, Kershaw MH, Smyth MJ, Trapani JA (2010). Gene-modified T cells as immunotherapy for multiple myeloma and acute myeloid leukemia expressing the Lewis Y antigen. Gene Ther.

[29] Ross JF, Wang H, Behm FG, Mathew P, Wu M, Booth R (1999). Folate receptor type beta is a neutrophilic lineage marker and is differentially expressed in myeloid leukemia. Cancer.

[30] Saito Y, Kitamura H, Hijikata A, Tomizawa-Murasawa M, Tanaka S, Takagi S (2010). Identification of therapeutic targets for quiescent, chemotherapy-resistant human leukemia stem cells. Sci Transl Med.

[31] Sarma A, Hazarika M, Das D, Kumar Rai A, Sharma JD, Bhuyan C (2015). Expression of aberrant CD markers in acute leukemia: A study of 100 cases with immunophenotyping by multiparameter flowcytometry. Cancer Biomark.

[32] Zhang F, Liu X, Chen C, Zhu J, Yu Z, Xie J (2017). CD244 maintains the proliferation ability of leukemia initiating cells through SHP-2/p27kip1 signaling. Haematologica.

[33] Kikushige Y, Miyamoto T (2015). Identification of TIM-3 as a Leukemic Stem Cell Surface Molecule in Primary Acute Myeloid Leukemia. Oncology.

[34] Krupka C, Kufer P, Kischel R, Zugmaier G, Bogeholz J, Kohnke T (2014). CD33 target validation and sustained depletion of AML blasts in long-term cultures by the bispecific T-cell-engaging antibody AMG 330. Blood.

[35] van Rhenen A, van Dongen GA, Kelder A, Rombouts EJ, Feller N, Moshaver B (2007). The novel AML stem cell associated antigen CLL-1 aids in discrimination between normal and leukemic stem cells. Blood.

[36] van Rhenen A, Moshaver B, Kelder A, Feller N, Nieuwint AW, Zweegman S (2007). Aberrant marker expression patterns on the CD34 + CD38- stem cell compartment in acute myeloid leukemia allows to distinguish the malignant from the normal stem cell compartment both at diagnosis and in remission. Leukemia.

[37] Gill S, Tasian SK, Ruella M, Shestova O, Li Y, Porter DL (2014). Preclinical targeting of human acute myeloid leukemia and myeloablation using chimeric antigen receptor-modified T cells. Blood.

[38] Kenderian SS, Ruella M, Shestova O, Klichinsky M, Aikawa V, Morrissette JJ (2015). CD33-specific chimeric antigen receptor T cells exhibit potent preclinical activity against human acute myeloid leukemia. Leukemia.

[39] Laborda E, Mazagova M, Shao S, Wang X, Quirino H, Woods AK (2017). Development of a chimeric antigen receptor targeting c-type lectin-like molecule-1 for human acute myeloid leukemia. Int J Mol Sci.

[40] Tashiro H, Sauer T, Shum T, Parikh K, Mamonkin M, Omer B (2017). Treatment of acute myeloid leukemia with T cells expressing chimeric antigen receptors directed to C-type lectin-like molecule 1. Mol Ther.

[41] Grimwade D, Hills RK, Moorman AV, Walker H, Chatters S, Goldstone AH (2010). Refinement of cytogenetic classification in acute myeloid leukemia: Determination of prognostic significance of rare recurring chromosomal abnormalities among 5876 younger adult patients treated in the United Kingdom Medical Research Council trials. Blood.

[42] Dohner H, Estey EH, Amadori S, Appelbaum FR, Buchner T, Burnett AK (2010). Diagnosis and management of acute myeloid leukemia in adults: recommendations from an international expert panel, on behalf of the European LeukemiaNet. Blood.

[43] Ehninger A, Kramer M, Rollig C, Thiede C, Bornhauser M, von Bonin M (2014). Distribution and levels of cell surface expression of CD33 and CD123 in acute myeloid leukemia. Blood Cancer J.

[44] Pemmaraju N, Sweet KL, Lane AA, Stein AS, Vasu S, Blum W, et al. Results of Pivotal Phase 2 Trial of SL-401 in Patients with Blastic Plasmacytoid Dendritic Cell Neoplasm (BPDCN). Blood*.* 2017; 130(Suppl 1)(1298): Accessed December 11, 2017. Retrieved from http://www.bloodjournal.org/content/130/Suppl_1/1298.

[45] Cellectis Reports Clinical Hold of UCART123 Studies (press release). Cellectis. Published on September 04, 2017. Retrieved from http://www.cellectis.com/en/press/cellectis-reports-clinical-hold-of-ucart123-studies/.

[46] Kikushige Y, Miyamoto T, Yuda J, Jabbarzadeh-Tabrizi S, Shima T, Takayanagi S (2015). A TIM-3/Gal-9 autocrine stimulatory loop drives self-renewal of human myeloid leukemia stem cells and leukemic progression. Cell Stem Cell.

[47] Jan M, Chao MP, Cha AC, Alizadeh AA, Gentles AJ, Weissman IL (2011). Prospective separation of normal and leukemic stem cells based on differential expression of TIM3, a human acute myeloid leukemia stem cell marker. Proc Natl Acad Sci USA.

[48] Roth CG, Garner K, Eyck ST, Boyiadzis M, Kane LP, Craig FE (2013). TIM3 expression by leukemic and non-leukemic myeloblasts. Cytom B Clin Cytom.

[49] Goncalves Silva I, Gibbs BF, Bardelli M, Varani L, Sumbayev VV (2015). Differential expression and biochemical activity of the immune receptor Tim-3 in healthy and malignant human myeloid cells. Oncotarget.

[50] Goncalves Silva I, Ruegg L, Gibbs BF, Bardelli M, Fruehwirth A, Varani L (2016). The immune receptor Tim-3 acts as a trafficker in a Tim-3/galectin-9 autocrine loop in human myeloid leukemia cells. Oncoimmunology.

[51] Wherry EJ (2011). T cell exhaustion. Nat Immunol.

[52] Schnorfeil FM, Lichtenegger FS, Emmerig K, Schlueter M, Neitz JS, Draenert R (2015). T cells are functionally not impaired in AML: increased PD-1 expression is only seen at time of relapse and correlates with a shift towards the memory T cell compartment. J Hematol Oncol.

[53] Eyquem J, Mansilla-Soto J, Giavridis T, van der Stegen SJ, Hamieh M, Cunanan KM (2017). Targeting a CAR to the TRAC locus with CRISPR/Cas9 enhances tumour rejection. Nature.

[54] Kugler M, Stein C, Kellner C, Mentz K, Saul D, Schwenkert M (2010). A recombinant trispecific single-chain Fv derivative directed against CD123 and CD33 mediates effective elimination of acute myeloid leukaemia cells by dual targeting. Br J Haematol.

[55] Kloss CC, Condomines M, Cartellieri M, Bachmann M, Sadelain M (2013). Combinatorial antigen recognition with balanced signaling promotes selective tumor eradication by engineered T cells. Nat Biotechnol.

[56] Lanitis E, Poussin M, Klattenhoff AW, Song D, Sandaltzopoulos R, June CH (2013). Chimeric antigen receptor T Cells with dissociated signaling domains exhibit focused antitumor activity with reduced potential for toxicity in vivo. Cancer Immunol Res.

[57] Chen C, Li K, Jiang H, Song F, Gao H, Pan X (2017). Development of T cells carrying two complementary chimeric antigen receptors against glypican-3 and asialoglycoprotein receptor 1 for the treatment of hepatocellular carcinoma. Cancer Immunol Immunother.

[58] Carey A, Edwards DKT, Eide CA, Newell L, Traer E, Medeiros BC (2017). Identification ofinterleukin-1 by functional screening as a key mediator of cellular expansion and disease progression in acute myeloid leukemia. Cell Rep.

