# Supplementary figures and images for: Coexpression profile of leukemic stem cell markers for combinatorial targeted therapy in AML
Source: Leukemia. 2018 Jun 26;33(1):64–74. doi: 10.1038/s41375-018-0180-3 (PMC6326956; doi:10.1038/s41375-018-0180-3)

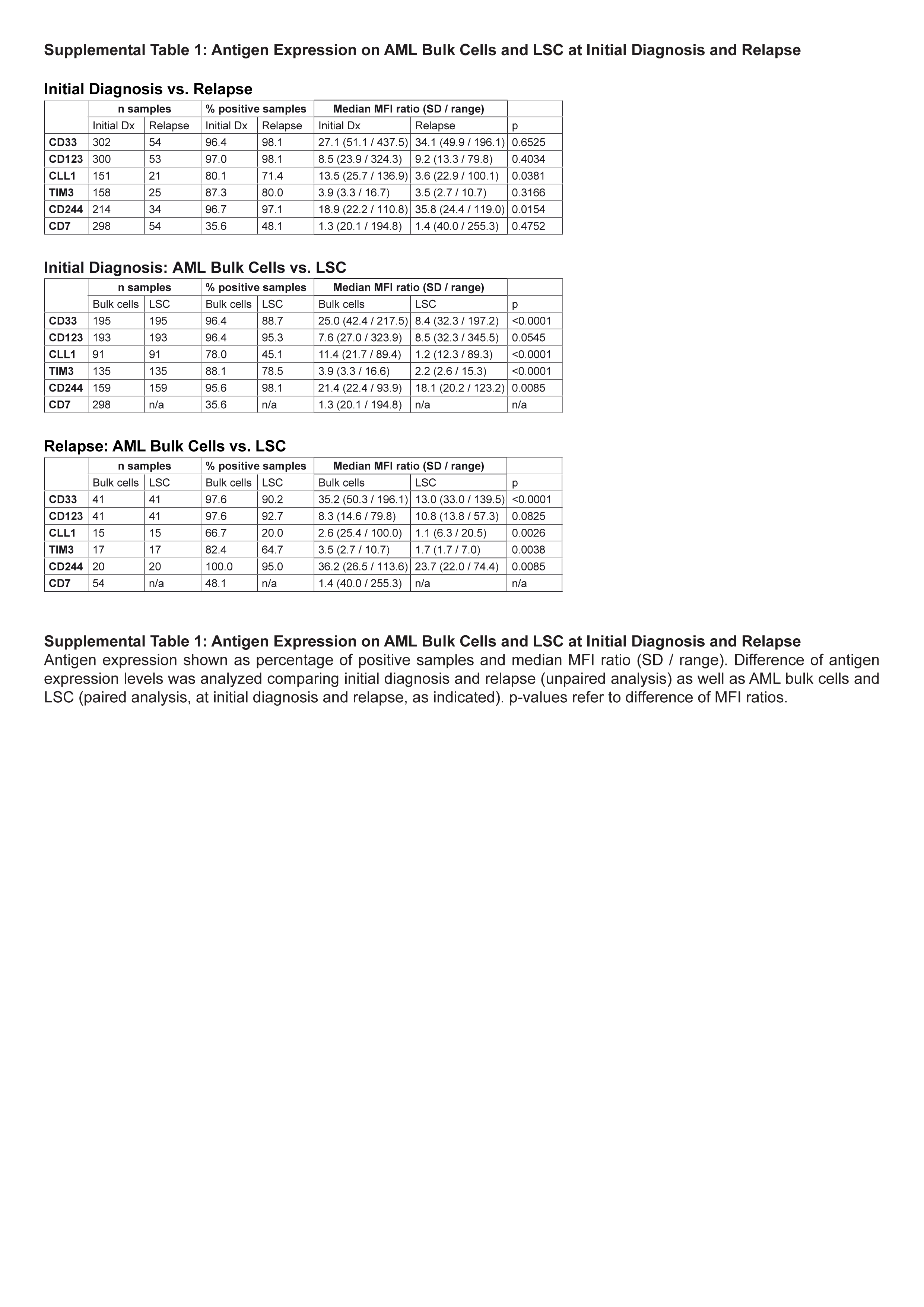

Supplement: Supplementary file 1 — Supplemental Table 1 [file 41375_2018_180_MOESM1_ESM.tif]

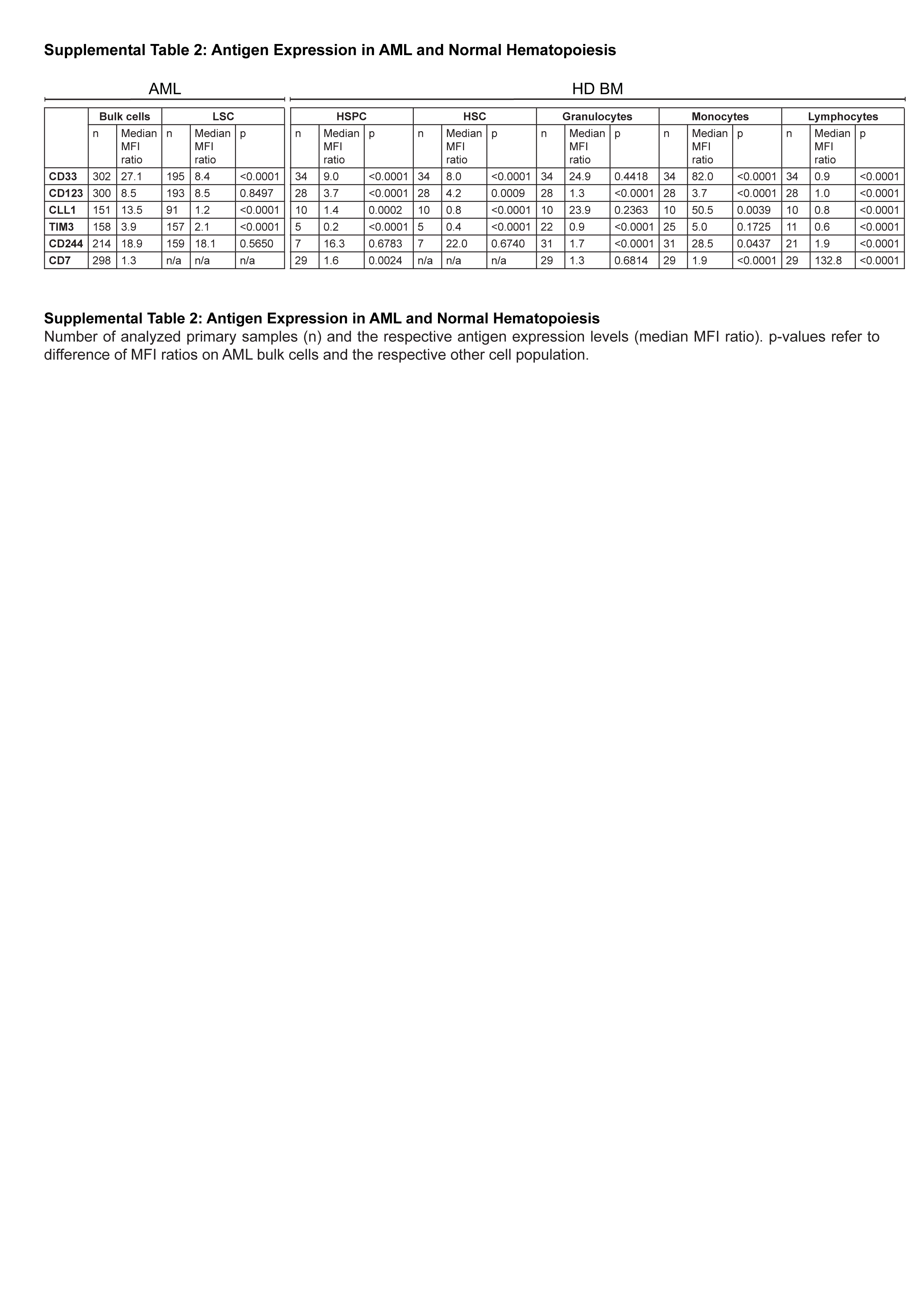

Supplement: Supplementary file 2 — Supplemental Table 2 [file 41375_2018_180_MOESM2_ESM.tif]

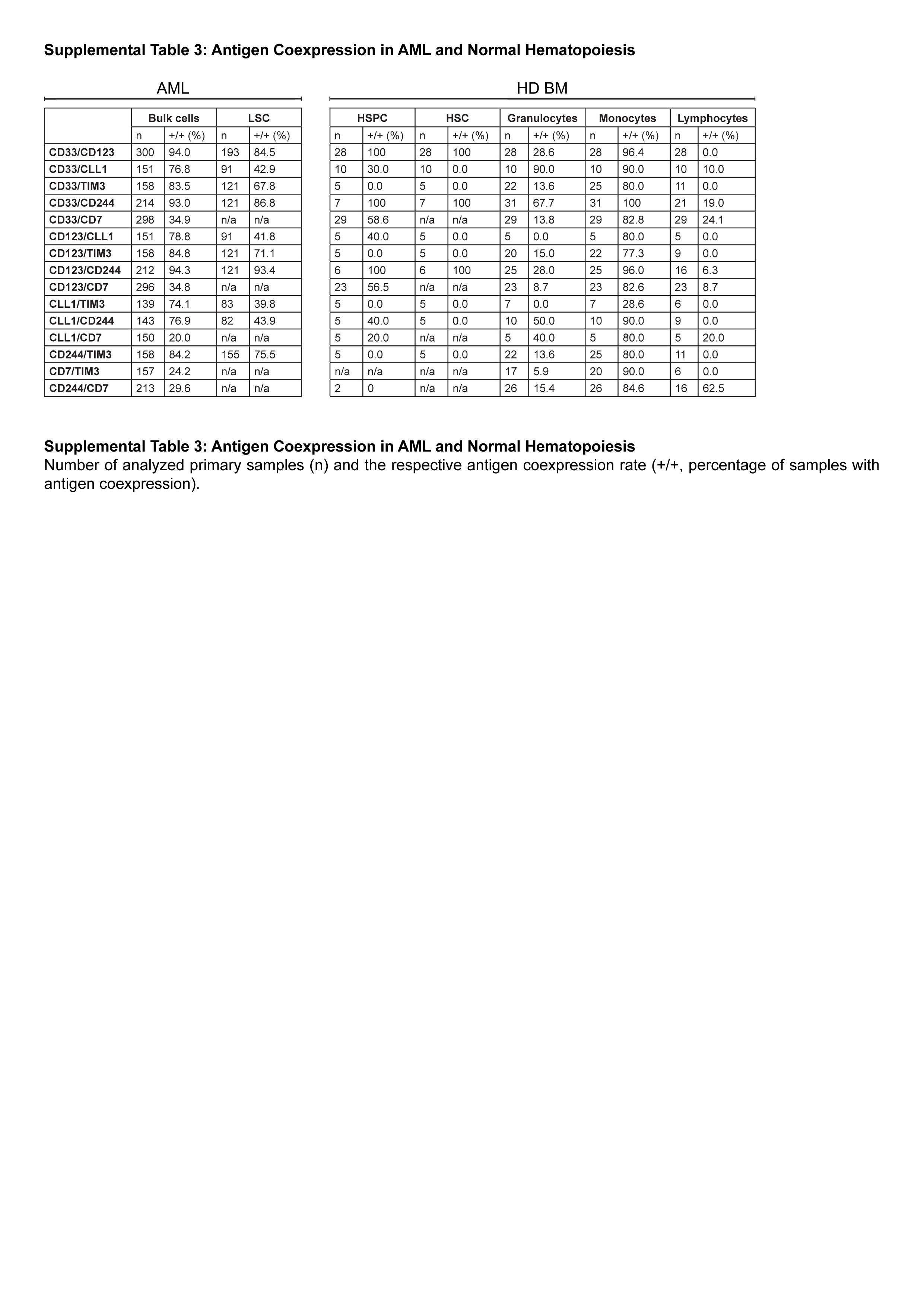

Supplement: Supplementary file 3 — Supplemental Table 3 [file 41375_2018_180_MOESM3_ESM.tif]

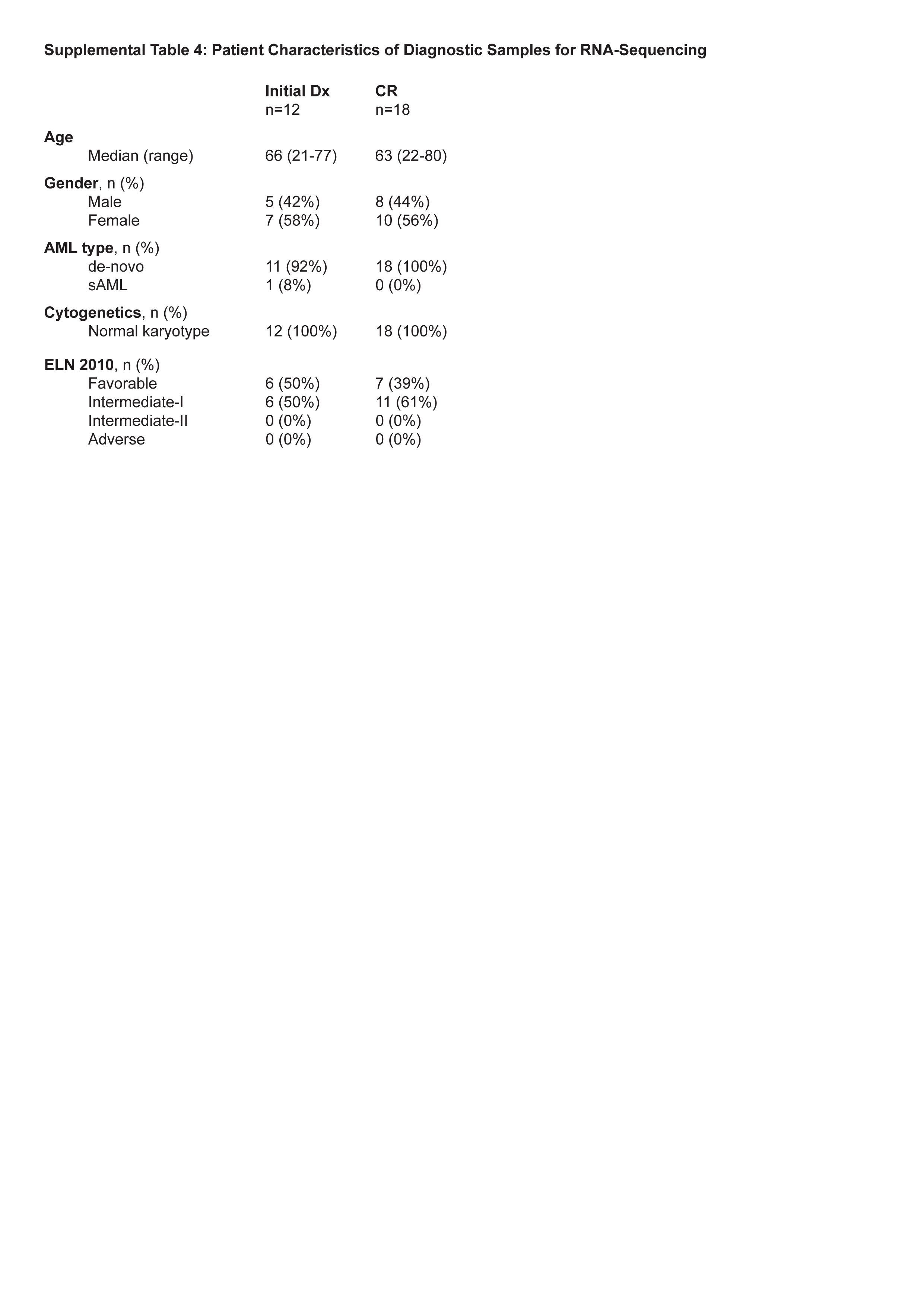

Supplement: Supplementary file 4 — Supplemental Table 4 [file 41375_2018_180_MOESM4_ESM.tif]

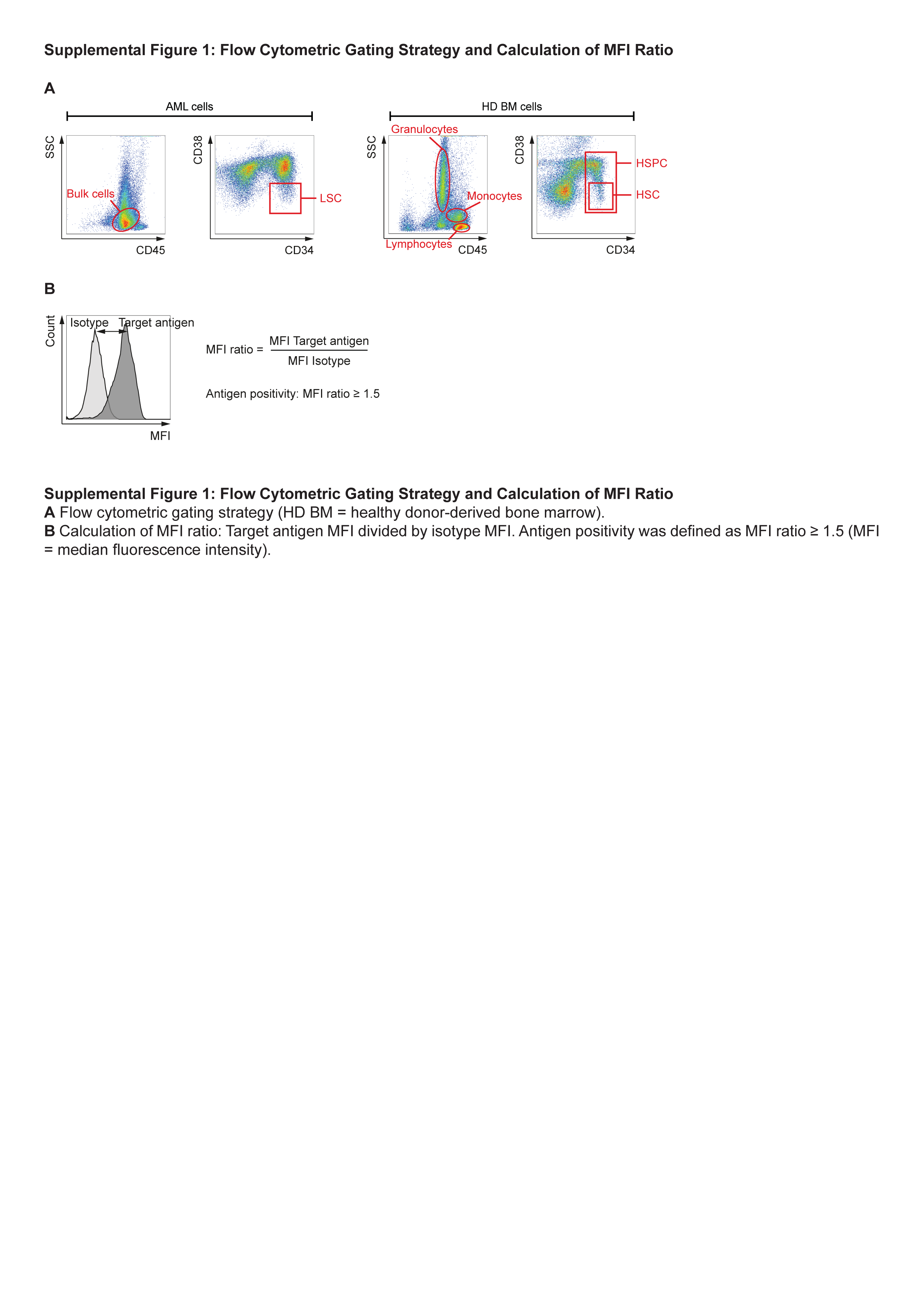

Supplement: Supplementary file 5 — Supplemental Figure 1 [file 41375_2018_180_MOESM5_ESM.tif]

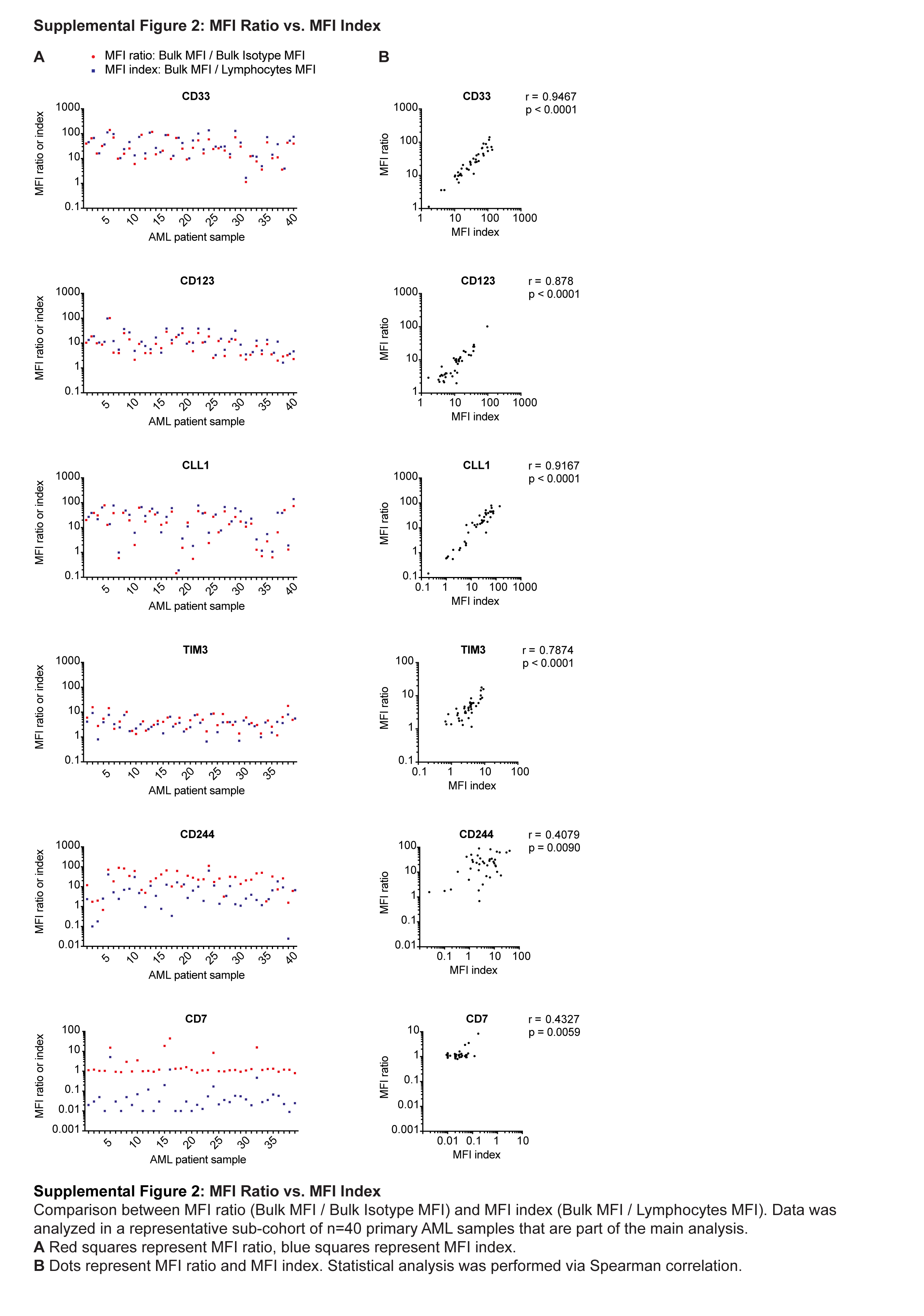

Supplement: Supplementary file 6 — Supplemental Figure 2 [file 41375_2018_180_MOESM6_ESM.tif]

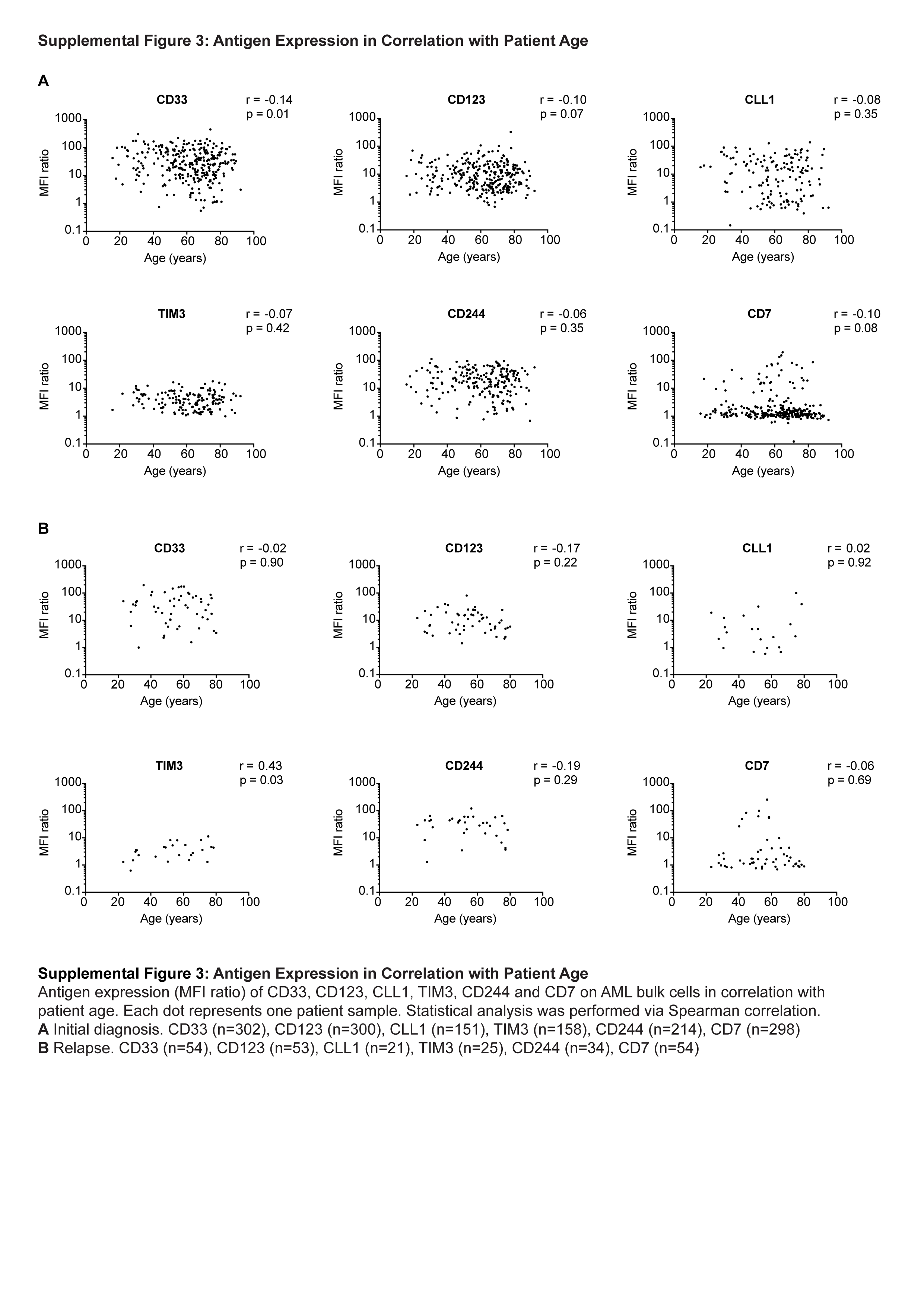

Supplement: Supplementary file 7 — Supplemental Figure 3 [file 41375_2018_180_MOESM7_ESM.tif]

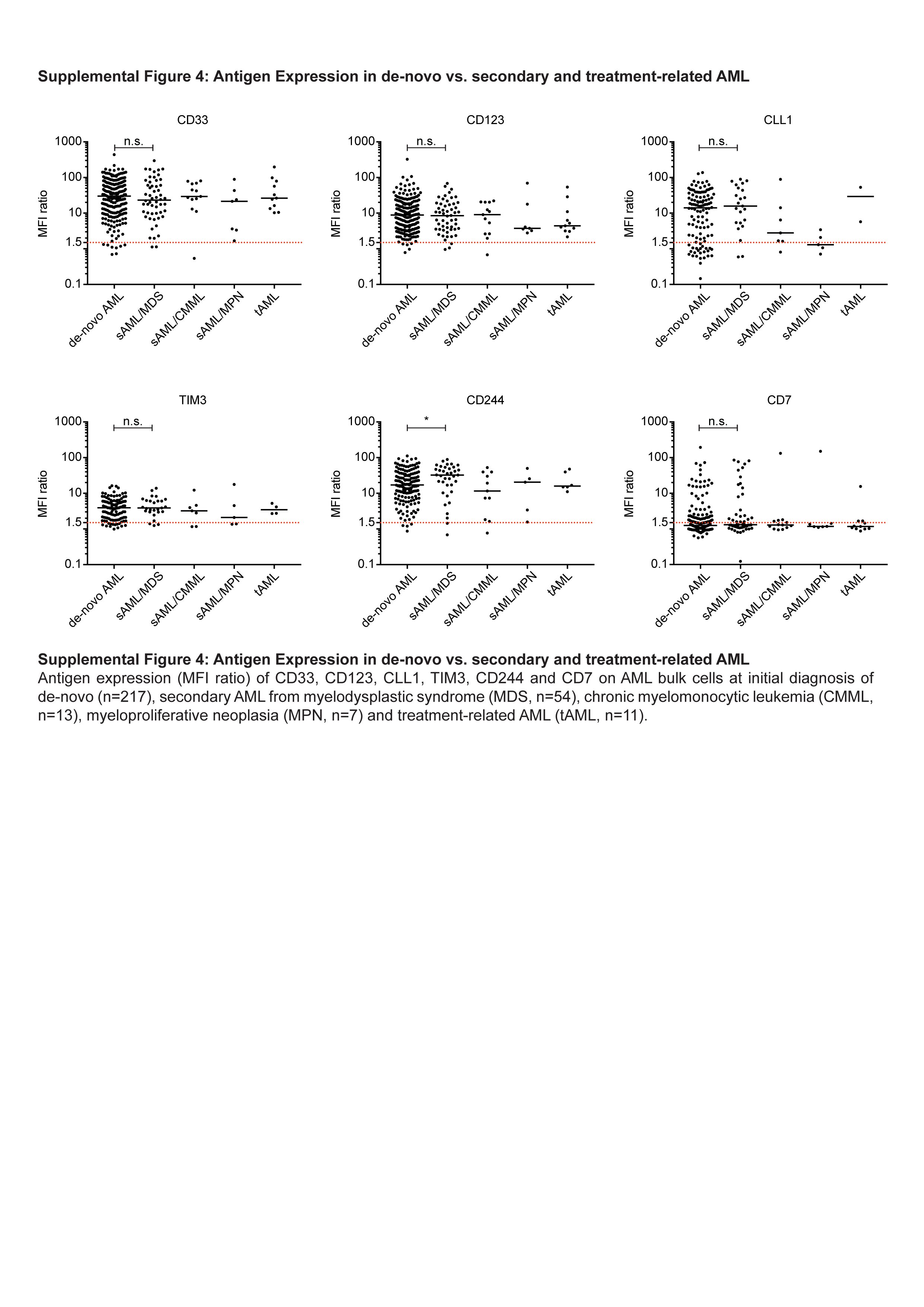

Supplement: Supplementary file 8 — Supplemental Figure 4 [file 41375_2018_180_MOESM8_ESM.tif]

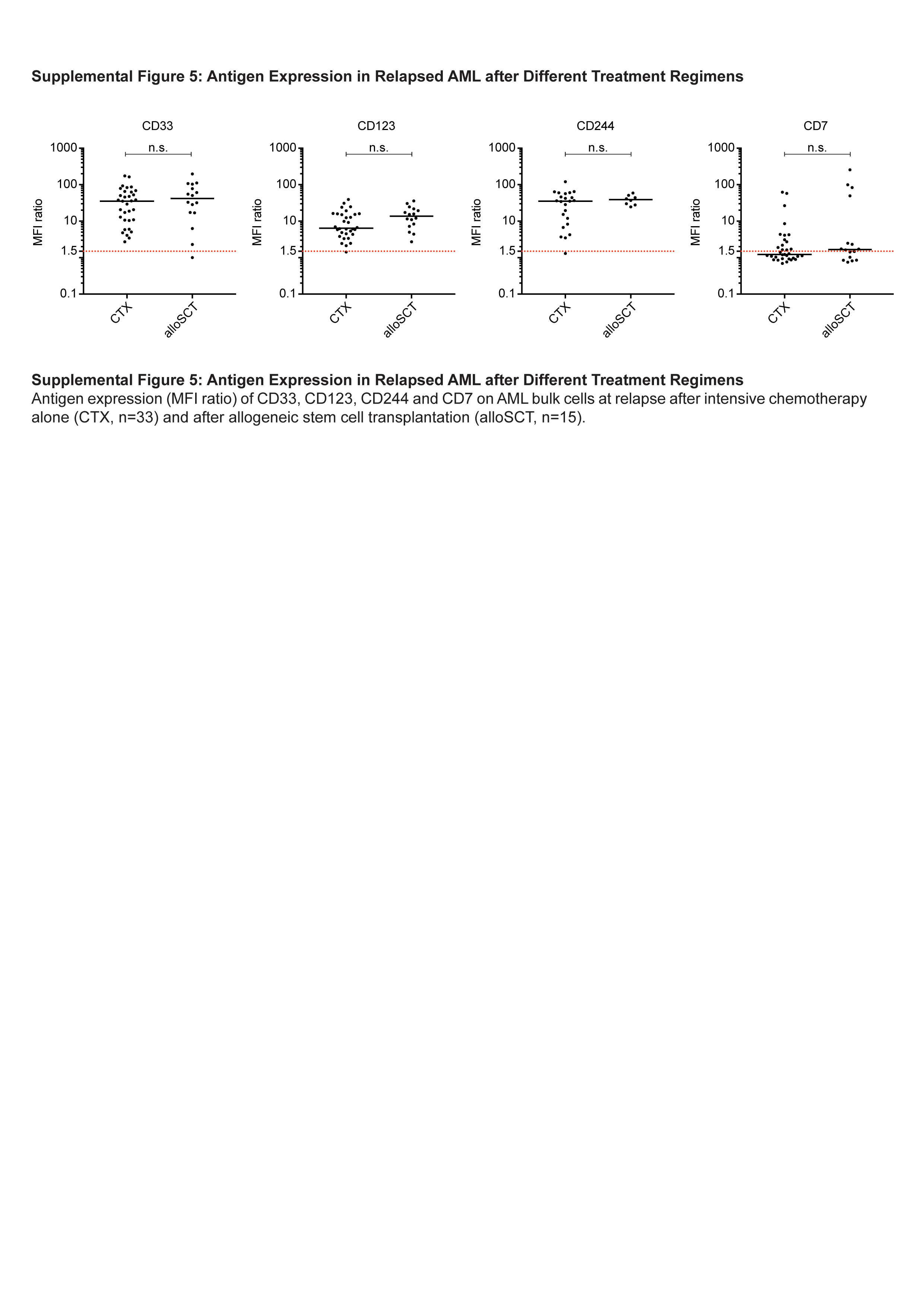

Supplement: Supplementary file 9 — Supplemental Figure 5 [file 41375_2018_180_MOESM9_ESM.tif]

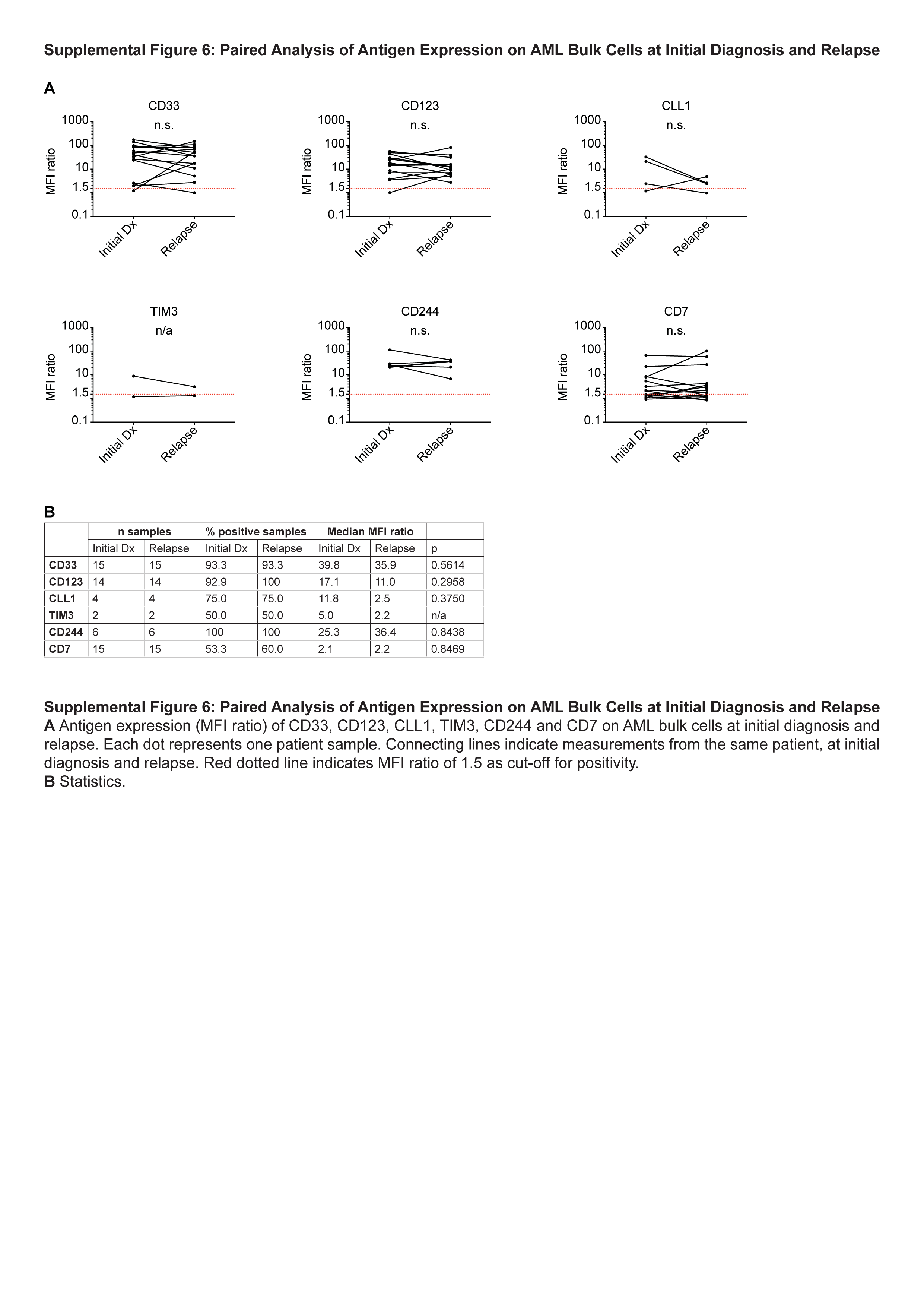

Supplement: Supplementary file 10 — Supplemental Figure 6 [file 41375_2018_180_MOESM10_ESM.tif]

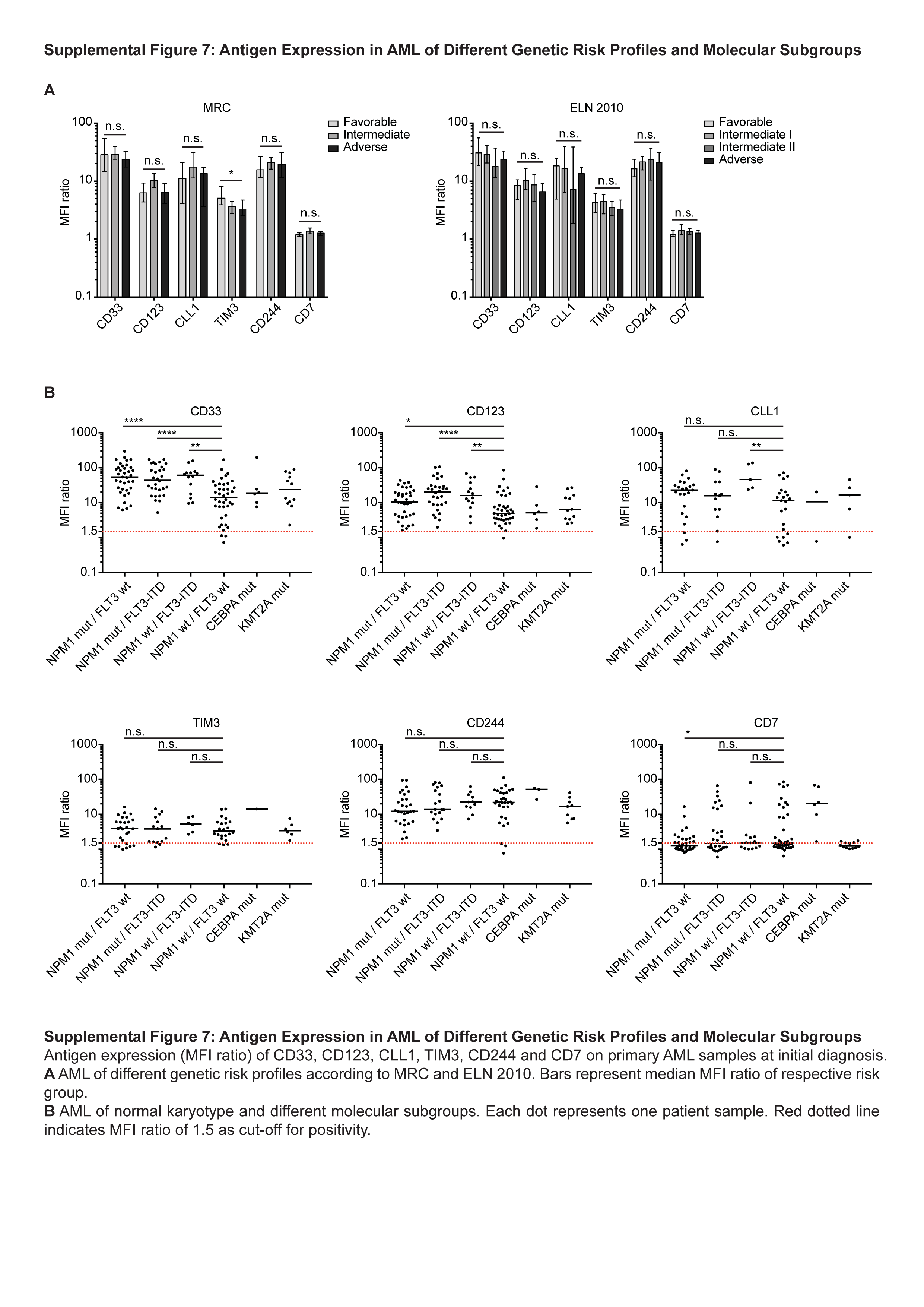

Supplement: Supplementary file 11 — Supplemental Figure 7 [file 41375_2018_180_MOESM11_ESM.tif]

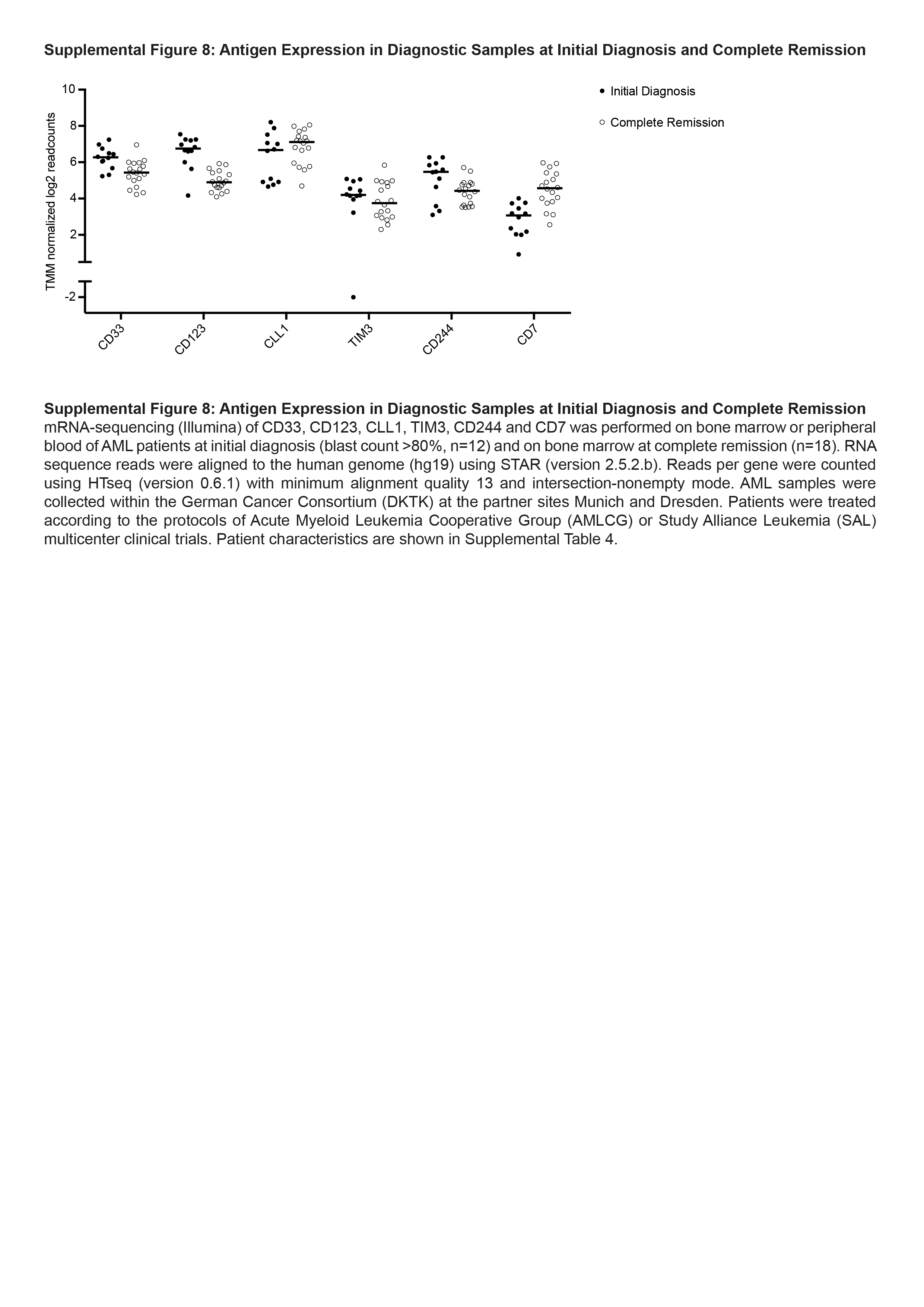

Supplement: Supplementary file 12 — Supplemental Figure 8 [file 41375_2018_180_MOESM12_ESM.tif]

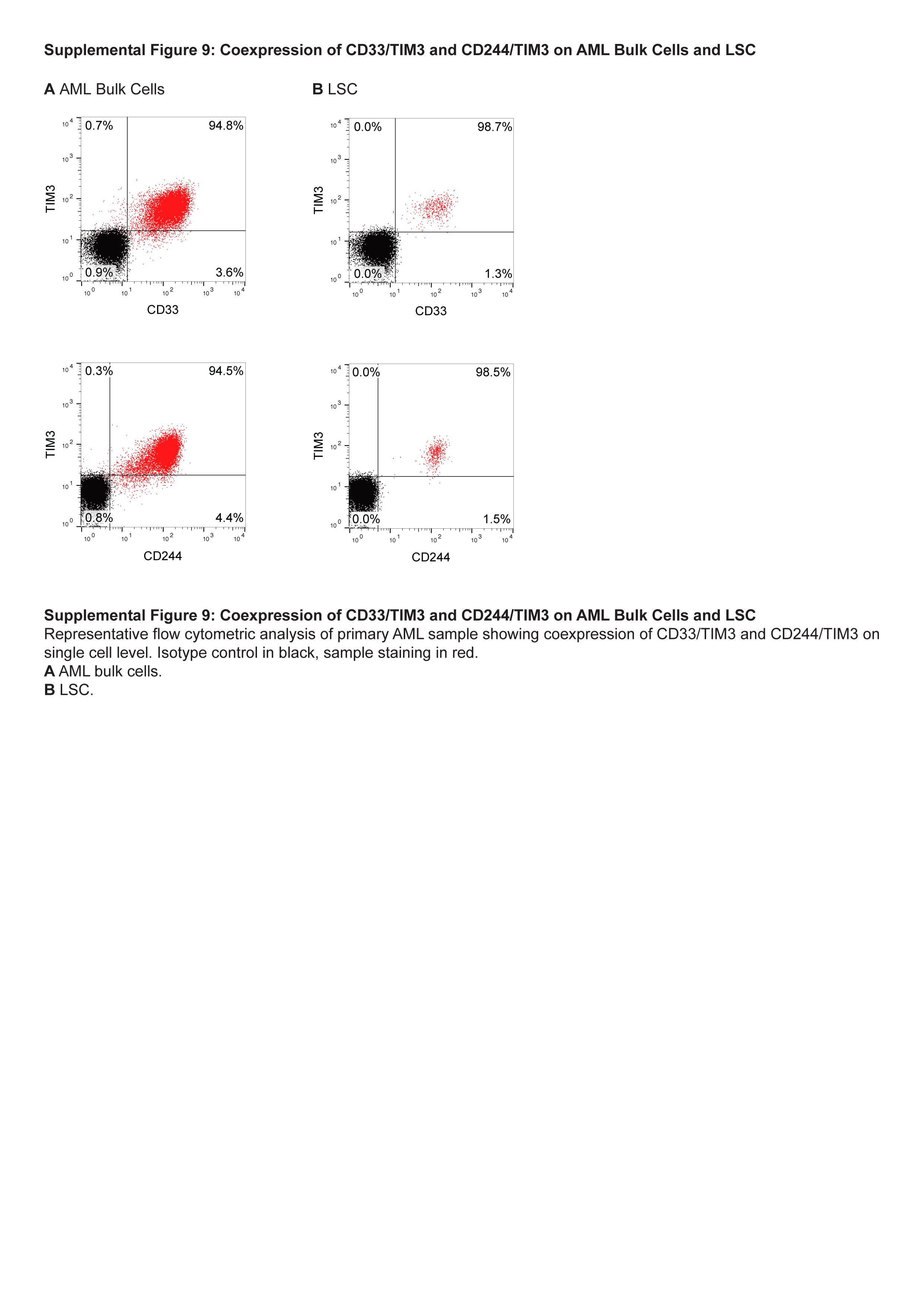

Supplement: Supplementary file 13 — Supplemental Figure 9 [file 41375_2018_180_MOESM13_ESM.tif]
